# The Vulcan salute sign: a non-sensitive but specific sign for Morton’s neuroma on radiographs

**DOI:** 10.1007/s00256-021-03851-3

**Published:** 2021-07-14

**Authors:** Julien Galley, Reto Sutter, Christoph Germann, Christian W. A. Pfirrmann

**Affiliations:** 1 Radiology, Balgrist University Hospital, University of Zurich, Zurich, Switzerland; 2 Radiology, HFR, University of Fribourg, Fribourg, Switzerland

**Keywords:** Musculoskeletal system, Radiography, Foot

## Abstract

**Objectives:**

To assess the value of the divergence of toes on conventional radiographs of the foot for diagnosing Morton’s neuroma.

**Methods:**

This retrospective case–control study was approved by the local ethics committee. In 100 patients with MRI-proven Morton’s neuroma 2/3 or 3/4 (study group) and 100 patients without (control group), conventional weight-bearing dorso-plantar view radiographs were evaluated for the subjective presence of interphalangeal divergence, called the Vulcan salute sign or V-sign, by two blinded, independent musculoskeletal radiologists. Interphalangeal angles (2/3 and 3/4) and intermetatarsal angle I/V were measured. The *t* test and chi-squared test were used to compare the groups. Diagnostic performance was calculated. Interobserver reliability was assessed using κ statistics and intraclass correlation coefficient (ICC).

**Results:**

The difference between the groups was significant (*P* < 0.05) regarding the presence of the V-sign, which was found in 30 of 100 patients with Morton neuroma and in 3 of 100 control patients, with a sensitivity of 30% and a specificity of 97%. The differences between interphalangeal angles were significant (*P* < 0.05) between the groups. The interphalangeal angle 2/3 mean values were 7.9° (± 4.8) for the study group vs 5.4° (± 2.6) for the controls; the 3/4 angle values were 6.5° (± 3.8) and 3.4° (± 2.5), respectively. There was no significant difference between the groups in the intermetatarsal angle I/V. Interobserver agreement was substantial for the V-sign, with a κ value of 0.78. The ICC was excellent concerning angle measurements, with all values ≥ 0.94.

**Conclusion:**

The Vulcan salute sign on conventional radiographs is specific for Morton’s neuroma.

## Introduction

Morton’s interdigital neuroma refers to a neuropathy of the interdigital nerve and is a common cause of forefoot pain [1]. Repetitive trauma and compression of the nerve are believed to result in vascular changes, endoneurial edema, and excessive bursal thickening, leading to perineural fibrosis. Consequently, Morton’s neuroma is not considered a true neuroma but rather a reactive perineural fibrosis [2–4]. This condition commonly affects the middle-aged population and is seen more frequently in women: according to Latinovic et al., the 1-year incidence of Morton’s metatarsalgia is 50.2 (men)/87.5 (women) per 100,000 [5]. In 1876, Thomas Morton first described pain localized in the fourth metatarsophalangeal articulation [6]; however, the most common location is the third interspace, followed by the second interspace [7, 8]. The differential diagnosis of forefoot pain is broad and complementary imaging may be necessary [9, 10]. MRI and ultrasound are the best imaging methods for the evaluation of the soft tissues of the forefoot and the assessment of Morton’s neuroma [11–13].

However, conventional radiography is still essential and often the first-line exam in evaluating metatarsalgia [14]. Radiographs are not currently considered to be of value for the evaluation of Morton’s neuroma, except for ruling out possible differential diagnoses such as a fracture. However, Morton’s neuroma may have a mass effect and therefore lead to divergence of the toes, clinically known as the Sullivan sign.

The purpose of the study was to evaluate the value of the divergence of the toes on conventional radiographs for the diagnosis of Morton’s neuroma.

## Materials and methods

This retrospective case–control study was approved by the local ethics committee.

### Study population

Two hundred patients presenting with forefoot pain were included in this study. All underwent conventional radiography and MR imaging of the forefoot for clinical indications between May 2014 and April 2019. All patients were older than 18 years. General written informed consent and permission to use the participants’ data for research purposes were obtained at the time of the X-ray examination.

#### Study group

The inclusion criteria for the study group were single interspace Morton’s neuroma (either 2/3 or 3/4) as demonstrated by MRI, and a time period of less than 2 months between X-rays and MRI. The exclusion criteria were multiple neuromas, previous surgery of the foot, recent fracture (< 2 months), advanced degenerative changes of the forefoot (defined as narrowing of the joint space), toe deformities (hammer toe, claw toe, mallet toe), and hallux valgus (defined as metatarsophalangeal angle > 15°).

#### Control group

Inclusion criteria for the control group were the absence of Morton’s neuroma as demonstrated by MRI and a time period of less than 2 months between X-rays and MRI. The exclusion criteria were the same as for the study group.

### Imaging

Conventional radiographs of the foot were taken from the standard weight-bearing dorsal-plantar (DP) view with the central ray directed over the middle of the third metatarsal. The X-ray tube was angled 15° cranially.

MR exams were performed on several MRI scanners: 1.5-Tesla units (MAGNETOM Avanto, Siemens Healthcare, Erlangen, Germany; OPTIMA 430, GE Healthcare, Waukesha, USA) or a 3-T unit (MAGNETOM Skyra, Siemens Healthcare, Erlangen, Germany) depending on availability. The details of the MR forefoot varied slightly between the different MR units. However, the protocol always included the following sequences: T1-weighted coronal (perpendicular to the metatarsal bones), T2-weighted coronal, and STIR axial images (parallel to the metatarsal bones). Morton’s neuroma was defined as a mass in the intermetatarsal space, equal or greater than 5 mm in diameter in the coronal plane [15], with low signal intensity in T1- and T2-weighted coronal MR images [11].

### Analysis of radiographs

**Fig. 1:**
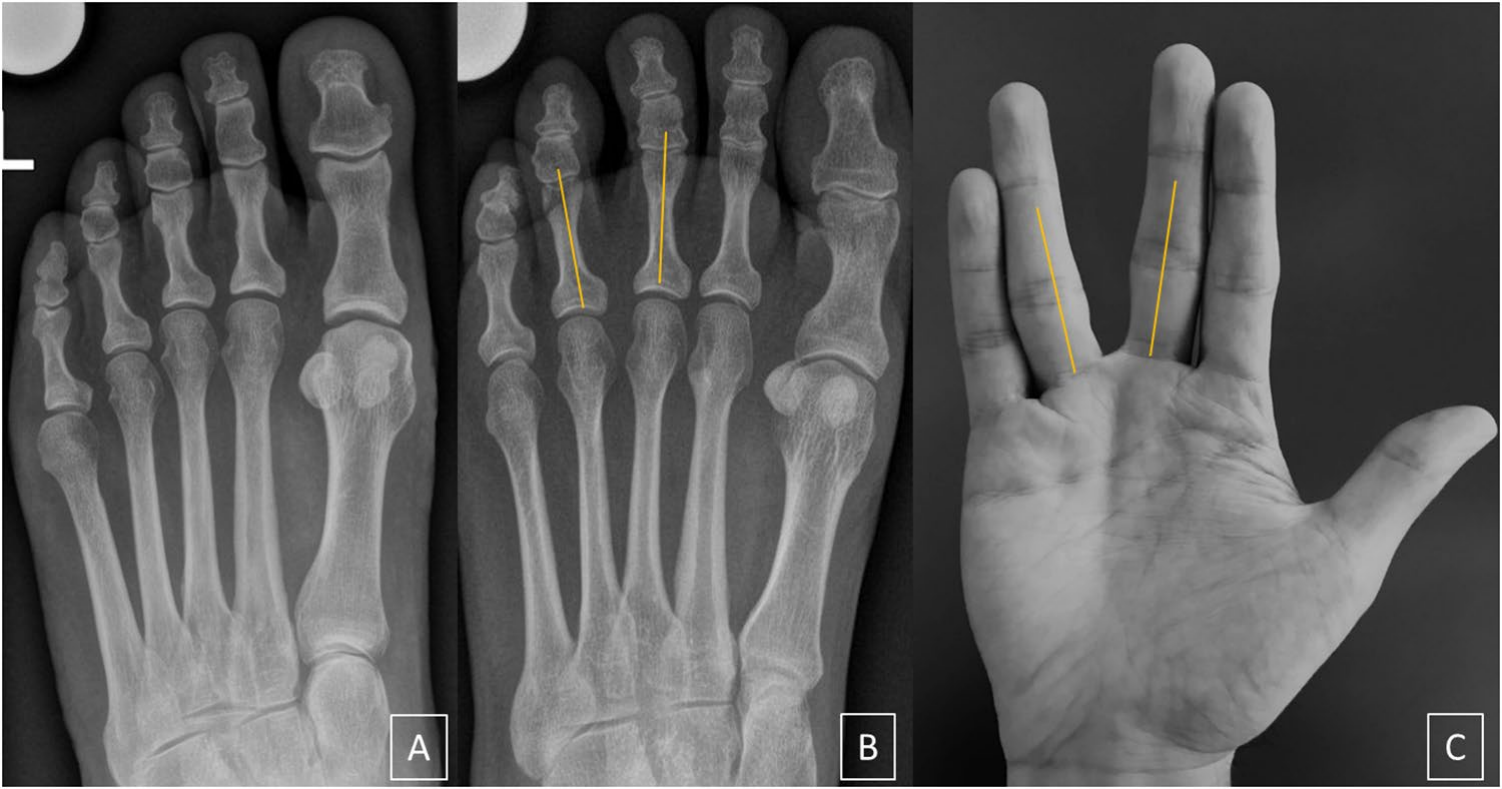
**A** Negative Vulcan salute sign in a 50-year-old woman from the control group. **B** Positive Vulcan salute sign (V-sign) of the 3/4 interspace in a 46-year-old woman with Morton’s neuroma. **C** “Vulcan salute” from one of the authors

**Fig. 2:**
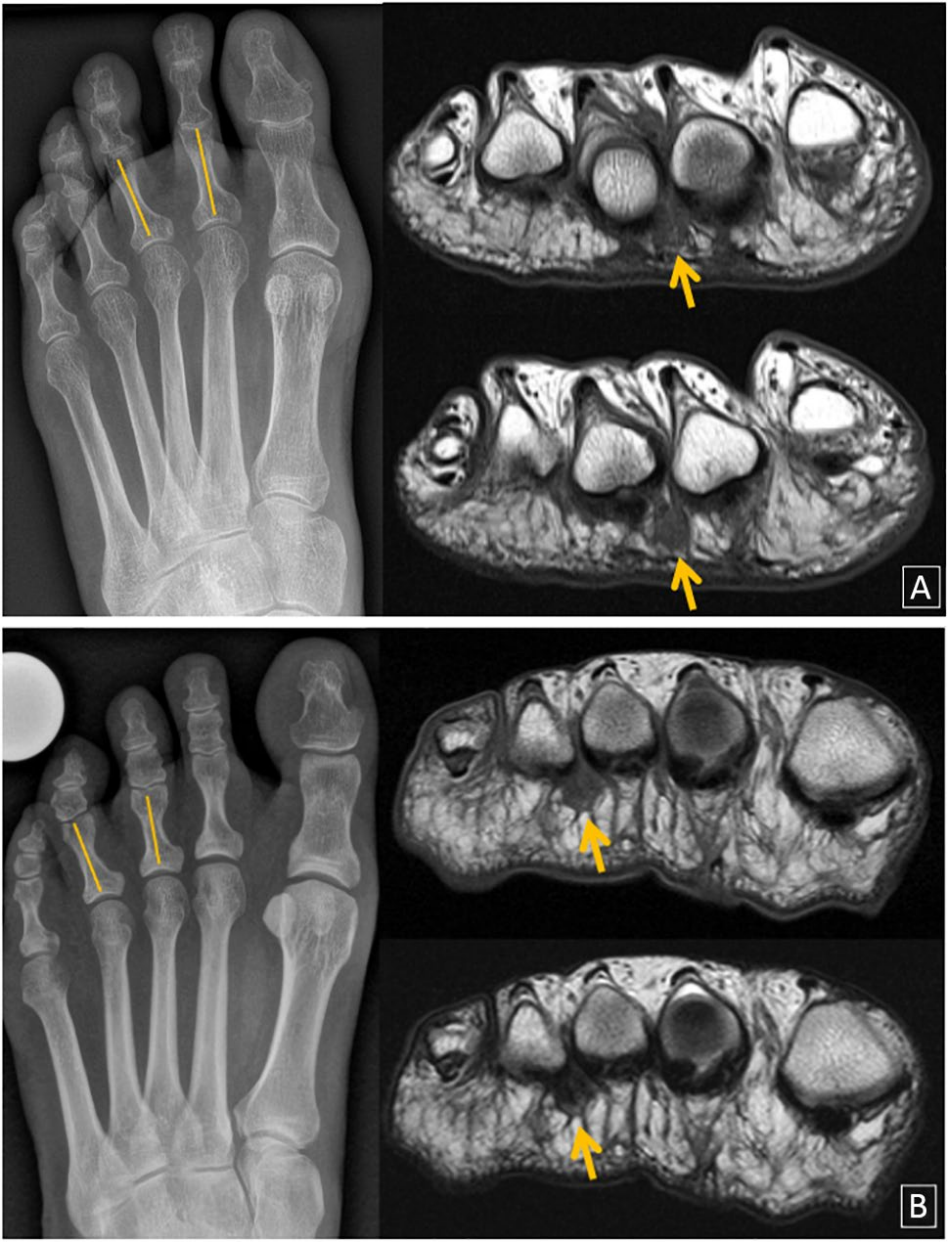
Positive V-sign. **A** Fifty-two-year-old woman with positive 2/3 V-sign on radiograph (left). Coronal T1-weighted (up) and T2-weighted (down) spine-echo MR images demonstrate the presence of a typical T1/T2 hypointense Morton’s neuroma (orange arrow). **B** Forty-nine-year-old woman with positive 3/4 V-sign on conventional radiograph (left) and the corresponding MR images (right) showing Morton’s neuroma (orange arrow)

**Fig. 3:**
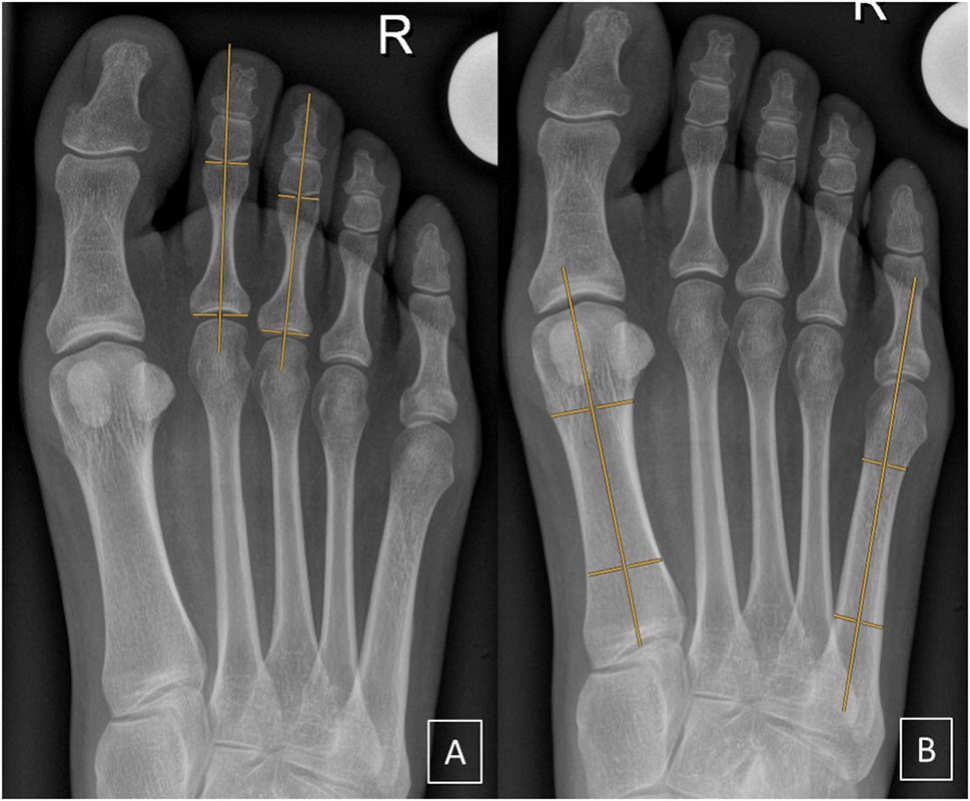
Angle measurements demonstrated on radiographs in a 27-year-old woman from the control group. **A** Interphalangeal angle 2/3. **B** Intermetatarsal angle I/V

Radiographs were evaluated independently by two fellowship-trained musculoskeletal radiologists (6 and 7 years of experience in musculoskeletal imaging) who were blinded to the clinical data and the diagnosis. The radiographs were presented in a randomized fashion. Each reader was first asked to assess the presence (yes/no) of the Vulcan salute sign (V-sign) of the interphalangeal 2/3 and 3/4 interspaces. The V-sign was defined as a subjective impression of proximal interphalangeal divergence (Figs. 1 and 2), referring to the clinical Sullivan’s sign. Then, the 2/3 and 3/4 interphalangeal angles were measured as follows: two transverse lines crossing the two outer borders of the articular surface (proximal and distal) were drawn; the point equidistant to the articular borders was defined as the central reference point (proximal and distal) to draw the final axis (Fig. 3A).

As we hypothesized that digital divergence could be influenced by the width of the forefoot, the intermetatarsal angle between the first and fifth metatarsal was also evaluated. It was defined as proposed by Coughlin et al. [16]: two references points located on a transverse line perpendicular to the longitudinal axis at a point equidistant from both cortical, proximal, and distal located between 1 and 2 cm from the articular surface (except for the proximal reference point of the fifth metatarsal located at the height of the cortical crossing point of the fourth and fifth metatarsal; Fig. 3B).

### Statistical analysis

Statistical analysis was performed using SPSS (v23, IBM Corp., Somers, NY). The κ-statistic was used to assess interobserver agreement concerning the presence of the V-sign. κ value < 0 was defined as no agreement, 0–0.20 as slight, 0.21–0.40 as fair, 0.41–0.60 as moderate, 0.61–0.80 as substantial, and 0.81–1 as almost perfect agreement [17, 18]. For angle measurements, the interobserver agreement was evaluated by the intraclass correlation coefficient (ICC). The chi-squared test was used to compare the presence of the V-sign between the groups and the *t* test was used for comparison of angle measurements. Specificity, sensitivity and odds ratio were also evaluated for the presence of V-sign.

## Results

### Study population

Morton’s neuroma group consisted of 80 women and 20 men with a mean age of 45.1 (± 9.8) years. Twenty patients presented a neuroma in the second interspace (2/3) and 80 in the third interspace (3/4). The control group consisted of 75 women and 25 men with a mean age of 42.6 (± 11.4) years.

### Vulcan salute sign or V-sign

The frequency of the V-sign was highly significant between groups with and without Morton’s neuroma (*P* < 0.05). The detailed results are presented in Table 1. Concerning the 2/3 interspace, the sensitivity of the V-sign for Morton’s neuroma was 40% for both readers; the specificity was 98% for reader 1 and 97% for reader 2. For the 3/4 interspace, the sensitivity values were 28% and 31%; the specificity was 99% for both readers. The overall sensitivity of the V-sign for Morton’s neuroma was 30% for reader 1 and 33% for reader 2; specificity was 97% and 96%, respectively. The odds ratio of the V-sign in predicting Morton’s neuroma was 33/22 (reader 1/reader 2) for the 2/3 interspace and 38/45 for the 3/4, respectively.

**Table 1:** Frequency and statistical significance of the V-sign (2/3 and 3/4) for both readers. *Data are numbers of cases, with percentages in parentheses

V-sign 2/3					
	Morton’s neuroma 2/3 group (n = 20)		Control group (n = 100)		P value
V-sign	Present	Absent	Present	Absent	
Reader 1	8 (40)*	12 (60)	2 (2)	98 (98)	˂0.05
Reader 2	8 (40)	12 (60)	3 (3)	97 (97)	˂0.05
V-sign 3/4					
	Morton’s neuroma 3/4 group (n = 80)		Control group (n = 100)		P value
V-sign	Present	Absent	Present	Absent	
Reader 1	22 (28)	58 (73)	1	99 (99)	˂0.05
Reader 2	25 (31)	55 (69)	1	99 (99)	˂0.05

### Angle measurements

A significant difference (*P* < 0.05) was found between the groups for interphalangeal angles 2/3 and 3/4 for both readers. The results are presented in Table 2. The results from reader 1 were the following: the mean value from the 2/3 interphalangeal angle was 7.9° (± 4.8) for the 2/3 study group and 5.4° (± 2.6) for the control group. The 3/4 angle value was 6.5° (± 3.8) for the 3/4 study group and 3.4° (± 2.5) for the control group.

**Table 2:** Mean values from interphalangeal angle 2/3, interphalangeal 3/4 angle, and intermetatarsal angle I/V from each group for both readers. *Data are degrees with standard deviation in parentheses

Interphalangeal angle 2/3			
	Morton’s neuroma 2/3 group	Control group	P value
Reader 1	7.9 (± 4.8)*	5.4 (± 2.6)	˂0.05
Reader 2	7.7 (± 4.5)	5.1 (± 2.6)	˂0.05
Interphalangeal angle 3/4			
	Morton’s neuroma 3/4 group	Control group	P value
Reader 1	6.5 (± 3.8)	3.4 (± 2.5)	˂0.05
Reader 2	5.7 (± 3.8)	3.7 (± 2.5)	˂0.05
Intermetatarsal angle I/V			
	Morton’s neuroma 2/3 group/3/4 group	Control group	P value
Reader 1	24.4 (± 3.4)/24.7 (± 3.8)	24.3 (± 3.5)	0.9/0.5
Reader 2	24.2 (± 3.3)/24.6 (± 3.8)	24.0 (± 3.5)	0.8/0.2

For cases with a positive 2/3 V-sign, the mean 2/3 angle was 12.5° (± 3.0) for reader 1 and 12.5° (± 3.0) for reader 2. For the cases with a positive 3/4 V-sign, the mean 3/4 angles were 11.5° (± 1.9) and 10.3° (± 2.1) for readers 1 and 2, respectively.

No significant difference between the groups was found for the intermetatarsal angle (*P* ≥ 0.5).

### Interobserver agreement

For the determination of the presence of the V-sign, interobserver agreement was substantial with a κ value of 0.78. The intraclass correlation coefficient was excellent for interphalangeal 2/3, interphalangeal 3/4, and intermetatarsal I/V angle measurements with values of 0.95, 0.94 and 0.96, respectively.

## Discussion

This paper presents the evaluation of interphalangeal divergence in patients with Morton’s neuroma on conventional radiographs. Interphalangeal divergence, as defined by the presence of the Vulcan salute sign or V-sign, is very specific for Morton’s neuroma but has a low sensitivity. Therefore, the presence of a V-sign on a plain radiograph is highly suggestive for the presence of a Morton neuroma in this interspace. A normal radiograph, however, does not exclude a Morton neuroma. The diagnosis of Morton’s neuroma is usually made through patient history and physical examination. Patients typically describe a burning pain at the plantar aspect of the forefoot (which can radiate distally to the toes or proximally towards the leg) and tingling or numbness in the toe. The symptomatology is usually worse on weight-bearing and patients frequently report the sensation of “walking on pebbles” [1, 7, 19]. Different dedicated clinical tests have been described, with the best diagnostic accuracy for the “thumb index finger squeeze test,” which consists of reproducing pain by squeezing the symptomatic interspace between the tips of the index finger and the thumb [20]. In these clinically clear cases, additional radiological exams are not required for the diagnosis [21]. However, symptomatology can be non-specific and imaging may be needed for diagnosis. Conventional radiography is recommended as the first radiological procedure by the ACR Appropriateness Criteria for the evaluation of chronic foot pain [22]. Numerous bony abnormalities may be diagnosed such as fractures, Freiberg disease, osteoarthritis, or even tumors. On ultrasound, Morton’s neuroma appears as a well-defined hypoechoic ovoid mass and typical cases will be positive for the sonographic Mulder sign (lateral compression of the metatarsal heads together to induce a plantar dislocation of the neuroma) [12, 23]. The presence of Morton’s neuroma in MRI is best visualized in a T1-weighted spine-echo sequence as a hypointense mass in intermetatarsal space, and is most clearly observable in a prone position [11, 24]. Both ultrasound and MRI have been proven to be very sensitive and specific exams for diagnosing Morton’s neuroma, with a similar sensitivity around 90% [25], and a higher specificity for ultrasound of 88% vs 68% for MRI, according to some authors [13].

The mass effect of Morton’s neuroma can lead to a divergence of the toes, which is visible on conventional radiographs. Therefore, radiographs may be helpful in diagnosing Morton’s neuroma. Weishaupt et al. showed the variability from Morton’s neuroma’s localization with patient positioning in MR [24]. As the standard dorso-plantar radiographs are taken in the weight-bearing position, Morton’s neuroma is most likely in a dorsal location between the metatarsal heads and proximal phalanges with a maximal mass effect, which may cause the phalanges to diverge. This hypothesis could be assessed by comparing weight-bearing to supine radiographs.

Naraghi et al. previously evaluated digital divergence and found no statistically significant difference between Morton’s neuroma subjects and controls [26]. However, their methodology differed from ours. First, they defined Morton’s neuroma as a clinical symptom with ultrasound correlation, but no minimum size of Morton’s neuroma was required for inclusion. Secondly, the exclusion criteria were not the same. Third, the absence of Morton’s neuroma in the control group was not confirmed by imaging.

Size has been shown to be correlated with symptomatology and a diameter of 5 mm is frequently consider the minimum size to cause symptoms [15, 27, 28]. However, some studies show no correlation [26, 29]. It is important to note that the purpose of our study was not to assess symptomatology but rather divergence. And if we accept the theory of a mass effect, size would make the difference.

Our study has the limitation that all of the patients who were included in the study underwent an MRI for clinical indications and that the exclusion criteria were broad. This may not represent a general population, leading to a possible selection bias.

In conclusion, the presence of the Vulcan salute sign or V-sign is highly specific for diagnosing Morton’s neuroma.

## Abbreviations

DP: Dorso-plantar
ICC: Intraclass correlation coefficient
MRI: Magnetic resonance imaging
